# Prevalence and predictors of patient no-shows to outpatient endoscopic procedures scheduled with anesthesia

**DOI:** 10.1186/s12876-015-0358-3

**Published:** 2015-09-30

**Authors:** Jennifer T. Chang, Justin L. Sewell, Lukejohn W. Day

**Affiliations:** 1 Division of Gastroenterology, Department of Medicine, University of California, San Francisco, CA USA; 2 Division of Gastroenterology, Department of Medicine, San Francisco General Hospital and Trauma Center, San Francisco, CA USA

**Keywords:** Endoscopy, Health outcomes, Quality, Health disparities

## Abstract

**Background:**

Demand for endoscopic procedures scheduled with anesthesia is increasing and no-show to appointments carries significant patient health and financial impact, yet little is known about predictors of no-show.

**Methods:**

We performed a 16-month retrospective observational cohort study of patients scheduled for outpatient endoscopy with anesthesia at a county hospital serving the safety-net healthcare system of San Francisco. Multivariate logistic regression analysis was performed to evaluate associations between attendance and predictors of no-show.

**Results:**

In total, 511 patients underwent endoscopy with anesthesia during the study period. Twenty-seven percent of patients failed to attend an appointment and were considered “no-show”. In multivariate analysis, higher no-show rates were associated with patients with a prior history of no-show (odds ratio [OR] 6.4; 95 % confidence interval [CI], 2.4- 17.5), those with active substance abuse within the past year (OR 2.2; 95 % CI 1.4-3.6), those with heavy prescription opioids/benzodiazepines use (OR 1.6; 95 % CI 1.0-2.6) and longer wait-times (OR 1.05; 95 % CI 1.00-1.09). Inversely associated with patient no-show were active employment (OR 0.38; 95 % CI 0.18-0.81), patients who attended a pre-operative appointment with an anesthesiologist (OR 0.52; CI 0.32-0.85), and those undergoing an advanced endoscopic procedure (OR 0.43; 95 % CI 0.19-0.94).

**Conclusion:**

In a safety-net healthcare population, behavioral and social determinants of health, including missed appointments, active substance abuse, homelessness, and unemployment are associated with no-shows to endoscopy with anesthesia.

## Background

When patients miss appointments, the efficiency of healthcare is reduced and patients experience longer wait times for healthcare [1, 2]. Longer wait times in turn may reduce healthcare quality and patients who routinely miss appointments experience poorer health outcomes [3, 4]. Furthermore, appointment nonattendance is more common among underserved populations [5–9], which can produce a high degree of economic strain on a resource-scarce healthcare system. Because increased utilization of safety-net healthcare settings is anticipated due to Medicaid expansion through the Affordable Care Act, optimizing operational efficiency is critical [10–12]. The issue of missed appointments is particularly pressing within the specialty of gastroenterology given the supply–demand mismatch for gastroenterology care in the safety-net system [5, 13, 14].

Endoscopy is an important setting in which to examine attendance because of its limited availability among many patient populations and the substantial financial impact of appointment no-show by patients [15]. The burden of digestive diseases in the United States and demand for gastroenterology specialty care has increased over the past decade [16]. Further, demand for endoscopic colorectal cancer screening exceeds supply and missed outpatient appointments may further limit patient access to endoscopy [17, 18]. Few studies have examined factors contributing to patient non-attendance at endoscopy [19–22] and studies that address the complex socioeconomic barriers specific to the safety-net setting are limited [23]. Moreover, little is known about predictors of no-show amongst patients undergoing diagnostic and therapeutic endoscopic procedures, where its impact on personal health is potentially greater than in screening examinations.

At the same time, utilization of anesthesia services during gastroenterology procedures increased substantially over the past decade and were used in more than 30 % of procedures nationwide and as high as 59 % in the northeast in 2009 [24]. No-show to endoscopy will be especially costly as utilization of anesthesia services during endoscopy is projected to continue to increase [24]. Identifying patients at high risk of no-show for endoscopy and the barriers to attendance can facilitate the development of interventions to improve attendance. Reducing missed appointments could increase efficiency and availability of gastroenterology care, minimize the financial losses associated with no-show, and has the potential to improve patient outcomes.

In this study, we examine predictors of no-show amongst patients undergoing outpatient diagnostic and therapeutic endoscopy scheduled with anesthesia support at a county hospital in San Francisco.

## Methods

### Study design

We performed a retrospective observational cohort study from 1/1/2012 thru 4/30/2013 of patients scheduled for an appointment to undergo endoscopy with monitored anesthesia care/general anesthesia in the safety-net healthcare system of San Francisco, California.

### Study population

San Francisco General Hospital provides subspecialty care for the safety-net healthcare system of the City and County of San Francisco, which includes multiple primary care clinics run by the San Francisco Department of Public Health, and affiliated independent Federally Qualified Health Centers and Federally-Funded 300(h) Grantee Centers. Patients are ethnically diverse (23 % Caucasian, 17 % African American, 29 % Hispanic, and 23 % Asian/Pacific Islander), and many are immigrants. Payer source for outpatient encounters were 37 % uninsured (26 % of which were provided by Healthy San Francisco, a city and federally funded universal health access plan for residents of the City and County of San Francisco), 35 % Medi-Cal (California’s Medicaid Program), 17 % Medicare, and 11 % commercial payers or other sources [25]. Eight percent of patients undergoing endoscopy were homeless for at least part of the year preceding endoscopy. The SFGH Gastroenterology Division receives 5,300 referrals annually for a wide spectrum of GI-related conditions and performs over 4,200 endoscopic procedures per year, approximately 10 % of which are performed with anesthesia services.

### Patient referral and scheduling

Patients were referred via an internet-based, electronic referral program (eReferral) to the gastroenterology clinic by their primary care provider or a subspecialty clinic. Initially, patients were evaluated in the gastroenterology clinic by a trainee (supervised by an attending gastroenterologist), nurse practitioner, or an attending gastroenterologist, during which informed consent for a procedure with anesthesia was obtained. If an endoscopic procedure was indicated, patients were scheduled for endoscopy with moderate sedation or with monitored anesthesia care/general anesthesia. Indications for scheduling an endoscopy with anesthesia care included all advanced endoscopic procedures including endoscopic retrograde cholangiopancreatography, endoscopic ultrasound, and single balloon-assisted enteroscopy, as well as patients with significant cardiopulmonary medical conditions, history of heavy substance abuse (including alcohol), heavy use of prescription opioids or benzodiazepines, history of failed sedation during endoscopy, and psychiatric illness. The decision to schedule a patient with anesthesia support was determined by the attending GI physician involved with the initial evaluation of the patient. Patients scheduled to undergo a procedure with general anesthesia were sometimes arranged for a formally scheduled pre-operative clinic evaluation by an anesthesiologist. Endoscopy with anesthesia was performed every Friday and every other Tuesday. All patients received telephone reminders one week prior to their scheduled appointment by the GI nursing staff and one day before the procedure by operating room staff. Ninety-five percent of the study population had a telephone number. Patients were not financially penalized if they failed to attend their appointment.

### Data sources

Data were obtained from two sources: the electronic medical record at San Francisco General Hospital (Lifetime Clinical Record®), and the endoscopy scheduling administrative records (Provation®). Identifying information was removed from subject data, assigned unique identification numbers, and data was compiled electronically into a single database. No subjects were contacted for the purposes of this study.

The primary outcome was whether a patient attended their endoscopy appointment scheduled with anesthesia support. Patients whose appointments were rescheduled or canceled were considered “no-show” for their appointment if they failed to attend a rescheduled appointment within 6 months. Predictor variables of patient no-show to their appointment included covariates with potential significance based upon the findings of other studies of healthcare access and utilization [5, 8, 9, 19–23]. Demographic data included age, sex, self-reported primary language, self-reported race/ethnicity, immigrant status, socioeconomic status, history of substance abuse or psychiatric diagnoses, and insurance status. Major language categories were as follows: English, Spanish, Asian language (which included Cantonese, Mandarin, Tagolog, and Vietnamese), and other. Patients were considered to have active substance abuse if review of medical records revealed self-report of active substance abuse or positive drug toxicology test within 1 year of the pre-endoscopy GI clinic encounter. Heavy use of prescription opioids or benzodiazepines was defined as the reported use of prescription opioids or benzodiazepines for treatment of chronic pain, substance abuse, or psychiatric illness that was determined to be a hindrance to adequate moderate sedation by the evaluating clinician during the pre-endoscopy GI clinic encounter. Referring clinician was grouped into PCP (primary care provider), subspecialist, or GI self-referral which included procedures scheduled directly from a prior procedure. Non-symptom-driven procedure indications included asymptomatic iron deficiency anemia, positive fecal occult blood/fecal immunohistochemical test, history of adenomatous polyp or cancer, and family history of colon cancer. Screening colonoscopies were not routinely performed. Wait-time was defined as number of weeks from pre-endoscopy GI clinic, inpatient, or procedure encounter or time from a canceled appointment to the rescheduled endoscopy appointment. Included as a predictor variable was procedure type with esophagogastroduodenoscopy (EGD) and colonoscopy grouped as routine procedures and endoscopic ultrasound (EUS), endoscopic retrograde cholangiopancreatography (ERCP), and single balloon-assisted enteroscopy grouped as advanced procedures.

### Statistical analysis

The power calculation was based on an estimated baseline no-show rate of 20 %. Based on prior studies of predictors of attendance at endoscopy [19–23], an estimated effect size of 15 % was assumed. Consequently, using a (2-sided) Z 0.05, power of 0.80 and b Z 0.2 for sample size calculations, it was determined that 91 patients were required for each group (no-show and show).

Continuous data are presented as means with standard deviations, whereas categorical data are presented as numbers and proportions. We compared patients who attended or missed their endoscopy appointment using bivariate and multivariable statistical methods. For bivariate analyses, categorical variables were compared using χ-square tests, and continuous variables were analyzed using two-tailed t-tests and ANOVA. Statistically significant results are noted with a *P* value ≤ 0 .05. A multivariate logistic regression was then performed in a stepwise fashion. Initial variables included, age, sex, race, language, immigrant, employment, homelessness, active substance abuse, heavy use of prescription opioids or benzodiazepines, insurance, type of procedure, attendance at pre-operative appointment, history of no-show, type of procedure, and length of time between clinic/rescheduling and the endoscopy appointment (wait-time). Variables that were not statistically significant (*P* > 0.05) were removed from the regression in a forward stepwise fashion. All calculations were performed by using Stata 11.0 (Stata Corp, College Station, Tex).

### Ethical considerations

This study was approved by the University of California San Francisco Committee on Human Research (IRB number 13-11585), the General Clinical Research Center at San Francisco General Hospital. The need for informed consent was waived.

## Results

### Patient population

In total, 511 patients underwent endoscopy with anesthesia support during the study period. Three hundred seventy-three (73 %) patients attended their endoscopy appointment. Of these, 50 patients canceled and attended a subsequent appointment within 6 months of their initial appointment. One hundred thirty-eight (27 %) of patients failed to attend an endoscopy appointment and were considered “no-show”. In contrast, 214 of 1714 (12 %) patients who underwent endoscopy with moderate sedation on the same days during the study period failed to attend an endoscopy appointment.

Our study included a diverse patient population, with 30 % self-identifying as white, 34 % black, 17 % Hispanic, and 17 % as Asian. Thirty-two percent were immigrants and 77 % identified English as their primary language. Seventy percent were unemployed or retired and 12 % were homeless. In terms of insurance status, 30 % were uninsured, 26 % had Medicare, and 44 % had Medical (Table 1). Mean time wait-time for an endoscopy procedure was 9 weeks.

**Table 1:** Characteristics of patients who were scheduled for an outpatient endoscopic procedure between 01/01/2012 and 04/30/2013

Variable	Show	No-show	Total	*p*-value
Age				
Mean (SD)	55.7 (10.9)	54.5 (11.5)	55.4 (11.1)	0.28
Male sex, no. (%)	203 (54.4)	91 (65.9)	294 (57.5)	0.02
Race/Ethnicity^a^, no. (%)				
White	108 (29.0)	44 (31.9)	152 (29.8)	Reference
African American	110 (29.5)	64 (46.4)	174 (34.1)	0.13
Hispanic	73 (19.6)	13 (9.4)	86 (16.8)	0.02
Asian	72 (19.3)	13 (9.4)	85 (16.6)	0.02
Language^b^ , no. (%)				
English	273 (73.2)	122 (88.4)	395 (77.3)	Reference
Spanish	48 (12.9)	8 (5.8)	56 (11.0)	0.01
Asian language	42 (11.3)	6 (4.4)	48 (9.4)	0.01
Immigrant, no. (%)	138 (37.6)	24 (17.7)	162 (32.2)	<0.001
Employed, no. (%)	77 (24.6)	10 (8.6)	87 (19.6)	<0.001
Homeless, no. (%)	36 (9.7)	26 (18.8)	62 (12.1)	0.01
Active substance abuse, no. (%)	91 (24.4)	68 (49.3)	159 (31.1)	<0.001
History of psychiatric illness, no. (%)	146 (39.1)	49 (35.5)	195 (38.2)	0.45
History of opioids or benzodiazepine prescription, no. (%)	103 (27.6)	64 (46.4)	167 (32.7)	<0.001
Insurance, no. (%)				
Uninsured	124 (33.2)	31 (22.5)	155 (30.3)	Reference
Medicare	93 (24.9)	39 (28.3)	132 (25.8)	0.02
Medical	156 (41.8)	68 (49.3)	224 (43.8)	0.06
Attendance at preoperative appointment, no. (%)	180 (48.3)	44 (31.9)	224 (43.8)	0.001
Known prior history of endoscopy - no. (%)	227 (60.9)	73 (52.9)	300 (58.7)	0.11
Prior no-show to endoscopy, no. (%)	8 (2.1)	17 (12.3)	25 (4.9)	<0.001
Date, no. (%)				0.34
1/1/2012-4/31/2012	77 (20.6)	22 (15.9)	99 (19.4)	
5/1/2012-8/31/2012	94 (25.2)	31 (22.5)	125 (24.5)	
9/1/2012-12/31/2012	93 (24.9)	34 (24.6)	127 (24.9)	
1/1/2013-4/30/2013	109 (29.2)	51 (37.0)	160 (31.3)	
Type of Procedure, no. (%)^c^				<0.001
Routine procedure	275 (73.7)	129 (93.5)	404 (79.1)	
Advanced procedure	98 (26.3)	9 (6.5)	107 (20.9)	
Symptom-driven procedure, no. (%)	199 (53.4)	65 (47.1)	264 (51.7)	0.21
Referring clinician - no. (%)				0.16
PCP	252 (67.6)	105 (76.1)	357 (69.9)	
Specialist	32 (8.6)	10 (7.3)	42 (8.2)	
GI	89 (23.9)	23 (16.7)	112 (21.9)	
Wait-time in weeks, mean (SD)	8.7 (6.2)	10.9 (6.5)	9.3 (6.4)	<0.001

^a^14 patients (10 show, 4 no-show) reported “other” race/ethnicity

^b^12 patients (10 show, 2 no-show) spoke “other” languages

^c^“Routine” procedures included EGD and/or colonoscopy. “Advanced” procedures included ERCP, EUS, and/or single balloon enteroscopy

### No-show rates among patients scheduled for an endoscopic procedure with anesthesia

**Fig. 1:**
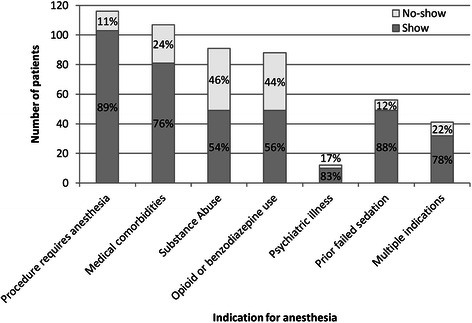
Number of patients stratified by the indication for use of anesthesia for their endoscopic procedure between 1/1/2012 thru 4/30/2013

There were significant bivariate differences between patients who missed and attended their endoscopy appointment (Table 1). There were statistically significant differences in no-show rates based on the indication for referral to anesthesia services for an endoscopic procedure (Fig. 1). Patients who required anesthesia services due to active substance abuse and those who were felt likely to fail moderate sedation due to heavy use of prescription opioids or benzodiazepines had the highest no-show rates at 46 % and 44 % respectively. This is in contrast to patients rescheduled with anesthesia due to failed sedation for which the no-show rate was 12 % and patients scheduled for advanced procedures that required general anesthesia for which the no-show rate was 11 %. In terms of patient characteristics, patients who missed their appointment were more likely to be men, homeless, or have active substance abuse, whereas patients who attended their appointment were more likely to be women, Asian or Hispanic, immigrants, employed, or undergoing an advanced procedure.

### Predictors of no-show for patients scheduled for an endoscopic procedure with anesthesia

There were a number of factors that predicted a patient not attending their endoscopy appointment in multivariable modeling (Table 2). Patients with a history of no-show had the greatest odds of not attending their endoscopy appointment (odds ratio [OR] 6.4; 95 % confidence interval [CI], 2.4-17.5). Also positively associated with a higher no-show rate were subjects with documented active substance abuse within the past year (OR 2.2; 95 % CI 1.4-3.6) and wait-time (OR 1.05; 95 % CI 1.00-1.09) with longer wait times associated with a greater no-show rate. We observed a strong trend toward positive association in subjects with heavy prescription opioids or benzodiazepines use (OR 1.6; 95 % CI 1.0-2.6). At the same time there were a number of factors inversely associated with no-show for outpatient general anesthesia endoscopic procedures. Patients with active employment had a 62 % lower odds of missing their procedure (OR 0.38; 95 % CI 0.18-0.81). Furthermore, patients who attended a pre-operative appointment with an anesthesiologist (OR 0.52; CI 0.32-0.85), and patients undergoing an advanced procedure (ERCP, EUS, or single balloon enteroscopy) (OR 0.43; 95 % CI 0.19-0.94) were less likely to miss their endoscopic procedure.

**Table 2:** Multivariable logistic regression of predictors for no-show to an outpatient general anesthesia endoscopic procedure

No-show variable	OR (95 % CI)	*P* value
Employed	0.4 (0.2-0.8)	0.012
Active substance abuse	2.2 (1.4-3.6)	0.001
Heavy use of prescription opioids or benzodiazepines^a^	1.6 (1.0-2.6)	0.053
Attendance at pre-operative appointment	0.5 (0.3-0.9)	0.009
Prior no-show for endoscopy	6.4 (2.4-17.5)	<0.001
Undergoing advanced procedure	0.4 (0.2-0.9)	0.035
Wait-time (weeks)	1.1 (1.0-1.1)	0.017

Note: Logistic regression was performed in a stepwise fashion. Initial variables included, age, sex, race, language, immigrant, employment, homelessness, active substance abuse, heavy use of prescription opioids or benzodiazepines, insurance, type of procedure, attendance at pre-operative appointment, history of no-show, type of procedure, and wait-time. Variables that were not statistically significant (*p* > 0.05) were removed from the regression in a forward stepwise fashion

*OR* odd ratio, *CI* confidence interval

^a^Heavy use of prescription opioids/benzodiazepines was defined as the reported use of prescription opioids or benzodiazepines that was determined to be a hindrance to adequate moderate sedation by the evaluating clinician during the pre-endoscopy GI clinic encounter

## Discussion

Utilizing anesthesia services for gastrointestinal endoscopy is increasingly common in the United States [23]. With rising healthcare costs and expanding healthcare coverage with the institution of the Affordable Care Act, cost-effective delivery of such services is paramount. Additionally, understanding predictors of no-show rates is critical in being able to deliver efficient endoscopy services. In this study, we observed an overall no-show rate of 27 % in outpatient endoscopy appointments scheduled with anesthesia support in a safety-net system. In contrast, we observed a much lower no-show rate of 12 % in outpatient endoscopy appointments with moderate sedation. There was high variability of no-show rates based on indication for anesthesia services with the highest rates of no-show seen in patients who required anesthesia services due to active substance abuse and heavy use of prescription opioids or benzodiazepines. Additionally, we discovered a number of predictors associated with no-show rates; specifically those who had previously missed their endoscopy appointment were more likely to not show for an endoscopic procedure whereas patients who had employment, attended a pre-operative anesthesia clinic, or were scheduled for an advanced endoscopic procedure were all more likely to show for their appointment.

Prior studies have shown high variability of attendance at endoscopy depending on practice setting. For example, rates of about 20 % have been described for colonoscopy nonattendance for follow-up of a positive FOBT in a Veterans Affairs setting [26] and rates as low as < 5 % for all open-access indications have been observed in an insured, high medical literacy population [20]. Traditionally, there is a high rate of nonattendance to appointments in the safety-net setting due to increased barriers to care [7–9]. Of the sparse data on this topic, no-show rates as high as 40 % have been documented for outpatient colonoscopies [23], which is higher than our observed rate of 27 %. A number of reasons may explain the high no-show rates we observed in the safety-net setting. Prior studies have suggested that race, limited-English proficiency, limited health insurance are all barriers to health care and attendance of clinic appointments [7–9, 27–29] and may also be barriers to attending scheduled procedures. However, the negative effects of health insurance and limited-English proficiency on attendance is not consistently observed in studies of subspecialty gastroenterology care, where health access via a primary care provider is already established [5]. In our study, we also did not observe any difference in attendance based on insurance status. It is important to note that most of the 30% of study subjects who were uninsured were enrolled in a city-wide universal health access program – Healthy San Francisco. Thus, our study findings may approximate healthcare in the safety-net setting under universal health insurance.

Data from our study have notable similarities and differences in comparison to other limited data on this subject. For example, we observed no difference in no-show rates based on whether or not a procedure was performed in a symptomatic versus asymptomatic patient. This finding is similar to a prior study where no difference was observed in diagnostic versus screening procedures [23]. Similar to prior studies, increased wait-time resulted in a higher no-show rate [5, 23]. Attendance of an additional pre-operative appointment were less likely to no-show, likely reflecting good appointment-keeping behavior, which has been previously shown to be a positive predictor of attendance to endoscopy [22]. However, notably there was a difference in no-show rates in patients undergoing a standard procedure such as EGD and colonoscopy compared to advanced procedures such as ERCP, EUS, and single balloon enteroscopy. This difference may be explained by increased attendance based on potential need for therapeutic intervention such as biliary obstruction or malignancy. The strength of our study compared with prior studies of endoscopy attendance [19–23], is that our study examined several important patient characteristics observed in clinical practice that likely adversely affect appointment keeping behavior. These include behavioral and social determinants of health including active substance abuse, homelessness, and unemployment. Our study underscores the importance and challenges of such barriers as they become most salient in a system with numerous interventions in place to address traditional barriers to non-attendance, including universal health-access, interpreting services, multi-language verbal and written instruction, telephone and mail reminders, and transportation home after a procedure.

The results from our study have important implications for patients scheduled for an outpatient endoscopic procedure with anesthesia that can help in the development and implementation of possible interventions. In particular, patients with a history of no-show to endoscopy were at much higher risk for no-show. For these select patients, it is worthwhile to investigate the reason for their failure to keep their prior appointment on an individual-level such as via a telephone call prior to scheduling a repeat appointment. In doing so, barriers for attendance to their procedure can be identified and on an individual-level can be addressed. Additionally, patients with active substance abuse are often socially marginalized and will require multidisciplinary care to address underlying psychosocial health problems. Further engaging these patient’s primary care providers, social workers, and hospital case management may be helpful in alleviating some of the unique challenges associated with healthcare delivery to these patients. Finally, stratifying patients’ likelihood of nonattendance, might allow for the judicious overbooking of endoscopy slots for patients at very high risk of nonattendance to maximize endoscopy unit operations efficiency.

There are several limitations to our study. First, the study was set in the safety-net setting, which serves a low income, underinsured, underserved patient population, many of which have limited English proficiency. While our study results may not be generalizable to some populations, it addresses a significant portion of the patient population that is likely to newly acquire health insurance with implementation of the Affordable Care Act given an established universal health access program for the uninsured in San Francisco. Second, information on co-pays required by patients was not collected. Thus, the effect of healthcare cost to the patient on attendance was not examined. Third, the study population was limited to subjects undergoing endoscopy with anesthesia services and did not include patients with moderate sedation. We chose to focus our study on endoscopy with anesthesia not only because we observe much higher rates of no-show in this population, but especially since anesthesia services are a limited and costly resource. Lastly, our study was retrospective in nature and a number of data abstracted was based on chart abstraction and this may have introduced a component of measurement bias.

## Conclusion

In summary, gastrointestinal endoscopy is a limited subspecialty resource throughout the United States and demand is expected to increase among vulnerable patient populations with institution of the Affordable Care Act. It is therefore vital that attendance at scheduled appointments be optimized in these patient groups. Even when applied to other, more privileged populations, our study underscores the importance of evaluating which patients fail to attend endoscopy, so that the efficiency and quality of subspecialty healthcare provided may be increased. Future studies should be aimed at targeted interventions that can mitigate the inefficient use of this expensive, limited resource in vulnerable patient populations.

## Abbreviations

CI: Confidence interval
OR: Odds ratio
GI: Gastroenterology
SFGH: San Francisco General Hospital and Trauma Center
